# Supplementary material for: WordCommentsAnalyzer: A windows software tool for qualitative research
Source: F1000Res. 2018 Sep 4;7:536. Originally published 2018 May 3. [Version 2] doi: 10.12688/f1000research.14819.2 (PMC6137411; doi:10.12688/f1000research.14819.2)
Supplement: Supplementary file 3 [file f1000research-7-17614-s0002.tgz › b2a79496-4ada-47c4-9682-a99ce867836d.zip!/Eight Iranian Health Journals/Journal of Health System Research _22.docx]

© Journal of Health System Research . This work is licensed under CC BY-NC 4.0 (https://creativecommons.org/licenses/by-nc/4.0/).

Volume & Issue: دوره 9، شماره 10؛ 1392

Title: بررسی اثر توام صدا و بارکاری بر روی تغییرات ضربان قلب در شرایط اسایش حرارتی: یک مطالعه آزمایشگاهی تحت کنترل

http://hsr.mui.ac.ir/index.php/jhsr/article/view/1067

مقدمه: صدا یکی از عوامل آزاردهنده در محیط زیست و زیان‌بخش در محیط کار است که توام شدن آن با فعالیت جسمانی در بسیاری از پست‌های کاری امری معمول است. هدف این مطالعه تعیین اثر مواجهه توام با شدت‌های مختلف صدا و بارکاری مختلف بر روی تغییرات ضربان قلب بود. روش‌ها: این مطالعه مقطعی- تجربی بر روی14 نفر از دانشجویان مرد با میانگین (انحراف معیار) سن 5/1 ± 0/ 23 سال، قد 2/ 5 ± 175 سانتیمتر، وزن 5/7 ± 7/6  کیلو گرم و نمایه توده بدنی 7/1 ±  9/21 کیلو گرم بر مجذور متر انجام شد. ضربان قلب در حالت استراحت(15دقیقه)، حالت نشسته(50 دقیقه)، فعالیت سبک (50 دقیقه)، فعالیت متوسط(50 دقیقه) و فعالیت سنگین(50 دقیقه)  تحت شرایط جوی کنترل شده در شرایط بدون مواجهه با صدا و در مواجهه با تراز های فشار صوت 65، 75، 85 و 95 دسی بل اندازه‌گیری شد. داده‌ها در نرم افرار SPSS نسخه 18 با آزمون تی- زوجی مورد تحلیل قرار گرفتند.  یافته‌ها: میانگین (انحراف معیار) ضربان قلب افراد در هنگام استراحت (5/7) 1/60 ضربان در دقیقه بود، در حالت نشسته، میزان افزایش ضربان قلب (4/2 ضربان در دقیقه) در مواجهه با تراز صدا 95 دسی‌بل در مقایسه با حالت بدون مواجهه با صدا معنی‌دار بود (032/P=). در فعالیت سبک، میانگین افزایش تعداد ضربان قلب در مواجهه با ترازهای 65، 75 و 95 دسی بل با میانگین ضربان قلب در حالت عدم مواجهه با صدا به ترتیب برابر با 1/2، 4/1 و 8/2 ضربه در دقیقه بود (01/ 0>P ). در فعالیت متوسط، میزان افزایش ضربان قلب در تراز های 65 ، 75 ، 85 و 95 دسی بل در مقایسه با حالت بدون مواجهه با صدا به ترتیب برابر با9/2 ، 8/3، 9/4 و 7/5 ضربه در دقیقه بود (001/0>P). در فعالیت سنگین، میانگین افزایش ضربان قلب در ترازهای 65، 75، 85 و 95 دسی‌بل در مقایسه با حالت بدون مواجهه با صدا به ترتیب برابر با 5/5، 4/8، 8/10 و 5/12 ضربه در دقیقه بود (001/0>P). نتیجه‌گیری: نتایج این مطالعه نشان داد که مواجهه با صدا در بارکاری مختلف و در شرایط دمایی مناسب موجب افزایش ضربان قلب می‌شود که روند افزایش ضربان قلب ناشی از مواجهه با صدا در حین انجام فعالیت‌های متوسط و خصوصا سنگین و در مواجهه با صدای بیشتر از حد مجاز 85 دسی‌بل زیادتر است

Title: مقایسه اثربخشی سخنراني و جزوه آموزشي برآگاهی، نگرش و رفتار تغذیه‌ای دانش‌آموزان شهر قم

http://hsr.mui.ac.ir/index.php/jhsr/article/view/1055

مقدمه: تغذيه نامناسب در بروز بيماري‌هايي گوناگون نظیر بيماري قلبی- عروقي، سرطان و ديابت تأثير به سزايي دارد. از آنجاكه اغلب رفتارهاي غلط بهداشتي ریشه در تجارب دوران كودكي و نوجوانی داشته و تغذیه سالم در دوران بلوغ تاثیر قابل توجهی بر رشد و سلامت انسان دارد، لذا هدف پژوهش حاضر بررسي تاثير دو روش آموزشي بر آگاهي، نگرش و رفتارهاي تغذيه‌اي دانش‌آموزان راهنمایی است. روش‌ها: در این مطالعه نيمه تجربي تاثیر دو روش آموزشی بر آگاهي، نگرش و رفتار تغذيه‌اي دانش‌آموزان مقطع سوم راهنمایی با استفاده از پرسشنامه‌اي شامل چهار بخش (‌مشخصات ‌فردی، آگاهي، نگرش و رفتار) مورد بررسي قرار گرفت. به روش خوشه‌ای دو مدرسه انتخاب و از هر مدرسه تعداد 110 دانش‌آموز پایه سوم (‌در مجموع 220 نفر) به صورت تصادفي در دو گروه آموزشی جزوه و سخنرانی قرار گرفتند. در ابتدا پرسشنامه توسط دانش‌آموزان به عنوان پیش‌آزمون تکمیل شد و سپس مداخله آموزشی در دو گروه در سه جلسه به اجرا درآمد و پس از سه ماه، همان پرسشنامه مورد آزمون مجدد قرار گرفت. داده‌های آماری توسط نرم‌افزار SPSS نسخه 16 با آزمون‌هاي T مستقل و زوجي و ضریب همبستگی بررسی شد. یافته‌ها: مداخلات آموزشی سبب افزايش معني‌داري در ميانگين نمرات آگاهي، نگرش و عملکرد دو گروه شد (‌05/0P<).  ميانگين تفاوت ايجاد شده در گروه سخنرانی در بخش آگاهی و نگرش به طور معنی‌داري بيش از گروه جزوه بود (‌05/0P<). نتیجه‌گیری: هر دو روش آموزشي، موجب افزايش سطح آگاهي، نگرش و رفتار تغذيه‌اي در نوجوانان شد. بنابراین توجه به برنامه‌هاي آموزشي جهت ارتقا رفتارهاي تغذيه‌اي امری ضروری است.

Title: تاثیر 3 فاکتور مداخله ارگونومی روی وضعیت بدنی و اختلالات اسکلتی عضلانی در کارکنان اداری (کاربران کامپیوتر)شرکت گاز

http://hsr.mui.ac.ir/index.php/jhsr/article/view/1008

مقدمه : بر اساس مطالعات انجام شده شیوع اختلالات اسکلتی عضلانی ناشی از کار (WMSDS )  در کاربران کامپیوتر بالا می‌باشد. هدف از این مطالعه تعیین تاثیر سه فاکتور مداخله ارگونومی روی وضعیت بدنی و اختلالات اسکلتی عضلانی در کارکنان اداری (کاربران کامپیوتر) شرکت گاز استان اصفهان بود. این سه مداخله شامل آموزش، ورزش و نصب نرم‌افزار بر روی سیستم کاربران کامپیوتر بود . روش‌ها : این مطالعه بر روی 98 نفر از کارمندان اداری شرکت گاز استان اصفهان، در سه فاز (بررسی وضعیت موجود – اجرای مداخلات و بررسی مجدد) به مدت 7 ماه انجام شد. در این مطالعه افراد شرکت‌کننده به چهار گروه تقسیم شدند (گروه آموزش، گروه ورزش، گروه نرم افزار و گروه کنترل). در این مطالعه 2 ماه قبل از مداخله‌ها و همچنین 3 ماه بعد از مداخلات اطلاعات مورد نظر جمع‌آوری گردید. برای این کار از پرسشنامه نوردیک جهت بررسی ناراحتی‌های اسکلتی عضلانی و از روش RULA (ارزیابی سریع اندام فوقانی) جهت ارزیابی وضعیت‌های بدنی استفاده شد و تاثیر هر یک از مداخلات بر روی ناراحتی‌های اسکلتی عضلانی بررسی شد. داده‌ها پس از جمع‌آوری توسط نرم افزار SPSS  وآزمون‌های مک نمار، تی‌تست و کای دو مورد تحلیل قرار گرفت. یافته‌ها: بر اساس ارزیابی انجام شده در فاز اول پروژه 5/53٪ از افراد بین 4 الی 8 ساعت با کامپیوتر کار می‌کردند و نوع کار بیشتر استاتیک بود. بر اساس این نتایج مشخص شد که آمار شیوع درد در نواحی کمر در سه ماهه گذشته و هفته گذشته یکسان است (5/53٪(. همچنین مشخص شد که 58٪ از افراد شرکت کننده شغل شان را عامل درد در ناحیه گردن و 39٪ از آنها شغل شان را عامل درد در ناحیه کمر می دانند. و در یکسال گذشته 24٪ از افراد به دلیل کمردرد، همچنین 2/15٪ از آنان به دلیل گردن درد از کار غیبت داشته‌اند. كاهش ناراحتي‌های اسكلتي عضلانی در گروه آموزش بعد از مداخله مشاهده گرديد كه اين كاهش با توجه به تست مك نمار در ناحيه کمر، گردن، زانو، مچ دست و پشت معني‌دار بود. (001/0P≤). نتایج حاصل از ارزیابی پوسچر به روش RULA  بعد از اجرای مداخلات نیز نشان داد که نمره ارزیابی پوسچر در گروه آموزش در سمت راست بدن 25 نمره و در سمت چپ بدن 20 نمره کاهش یافته است. بر اساس آزمون t تست این کاهش نمره معنادار گزارش شد، یعنی بعد از مداخله آموزش، ایستگاه کار بهبود پیدا کرد. ولی در سایر گروه‌ها تغییری مشاهده نشد. ناراحتي اسكلتي عضلانی گزارش شده توسط كاركنان گروه آموزش بعد از مداخله، مشاهده گرديد كه اين كاهش با توجه به تست مك نمار در ناحيه کمر، گردن، زانو، مچ دست و پشت معني‌دار بود (001/0P≤). نتیجه‌گیری: این بررسی نشان داد شیوع اختلالات اسکلتی عضلانی در گروه مورد مطالعه در نواحی کمر، گردن، زانو و پشت بالا بود و نیز آمار غیبت‌ها با آمار ناراحتی‌های اسکلتی عضلانی همخوانی داشت. علاوه بر این به نظر می‌رسد در گروه آموزش، با بکارگیری الگوهای مناسب دست و مچ دست و در نتیجه کاهش حرکات تکراری توانستند تغییرات مناسب را ایجاد نمایند. نکته جالب در این بود که صرف‌نظر از میزان اثر بخشی هریک از مداخلات، اکثر شرکت‌کنندگان با این مداخله‌ها موافق بودند و این نشان می‌دهد که افراد از وضعیت موجود ناراضی بوده و به دنبال ایجاد تغییر می‌باشند. به طور کلی یافته‌های ما نشان می‌دهد آموزش تنظیم صندلی و چیدمان صحیح محیط کار می‌تواند باعث کاهش مشکلات اسکلتی عضلانی شود.

Title: بررسی نقش عفو در سلامت و بهزیستی روانشناختی دانشجویان

http://hsr.mui.ac.ir/index.php/jhsr/article/view/991

مقدمه: فقدان سلامت از پيشرفت و توانايي انجام وظايف در فرد جلوگيري كرده و بهزيستي روانشناختي به عنوان رشد استعدادهاي واقعي فرد، موضوعات ارزشمندی برای مطالعه می‌باشند. پژوهش حاضر با هدف بررسي رابطه عفو با سلامت و بهزيستي روان‌شناختي در دانشجویان انجام شد. روش‌ها: اين مطالعه در مورد 243 دانشجو (113 پسر، 130 دختر) ساکن در خوابگاه دانشجویان دانشگاه تهران انجام گرفت. از شرکت‌کنندگان خواسته شد تا مقیاس عفو هارتلند، پرسشنامه سلامت عمومی و مقیاس بهزیستی روانشناختی را تکميل کنند. داده‌ها پس از جمع‌آوری با اسفاده از نرم‌افزار آماري SPSS نسخه 18 مورد تجزيه و تحليل قرار گرفت. به منظور بررسي رابطه بين متغيرها، ضريب همبستگي پيرسون و تحلیل رگرسیون مورد استفاده قرار گرفت. يافته‌ها: نتايج پژوهش نشان داد عفو با سلامت و بهزيستی روانشناختی همبستگي مثبت معنادار دارد. نتایج تحلیل رگرسیون نشان داد سلامت و بهزيستی روانشناختی توسط ابعاد عفو پیش‌بینی می‌شود. نتيجه‌گيري: ارزیابی و شناخت سلامت و بهزيستی روانشناختی و ارتباط اين ساختارهاي روانشناختي با عفو، می‌تواند رهنمودهای ارزشمندی را در زمینه تأمین بهداشت روانی برای دانشجویان به همراه داشته باشد. سلامت، بهزيستی روانشناختی و عفو نیازمند توجه بیشتری در تحقیقات بالینی هستند.

Title: بررسی عوامل مؤثر بر پیش‌بینی انجام ماموگرافی بر اساس تئوری رفتار برنامه‌ریزی در زنان شاغل 35 سال به بالا

http://hsr.mui.ac.ir/index.php/jhsr/article/view/983

مقدمه: سرطان پستان يكي ازشايع‌ترين بدخيمي‌هاي زنان و از علل اصلي مرگ و مير ناشي از سرطان در زنان در سراسر دنيا به حساب مي‌آيد. ماموگرافی به عنوان دقیق‌ترین روش تشخیص زودرس سرطان پستان می‌باشد و علیرغم اینکه ماموگرافی با تشخیص زودرس و تسریع در درمان، میزان مرگ و میر ناشی از سرطان پستان را کاهش داده است بسیاری از زنان ماموگرافی را انجام نمی‌دهند. مطالعه حاضر با هدف تعيين عوامل مؤثر بر انجام ماموگرافی بر اساس تئوری رفتار برنامه‌ریزی شده در زنان شاغل از 35 سال به بالا می‌باشد . روش‌ها: این مطالعه مقطعی، از نوع توصیفی تحلیلی می‌باشد که بر روی 320 زن شاغل در سال 1388 انجام گرفت. پس از جمع‌آوری اطلاعات به وسیله پرسشنامه محقق ساخته با بهره‌گیری از پرسشنامه  MBAQو کدگذاری آنها، آنالیز اطلاعات با استفاده از آزمون‌های آماری t-test، خی‌دو و آنالیز رگرسیون لجستیک صورت گرفت. یافته‌ها: نتایج حاصل از مطالعه نشان دادکه بین گروهی که ماموگرافی انجام دادند (27% از کل افراد مورد بررسی) و گروهی که انجام ندادند در میانگین نمرات هنجار های انتزاعی(001/0P<) ، قصد رفتار و درک کنترل رفتار (001/0P<) اختلاف معناداری وجود دارد. همچنین آنالیز رگرسیون لجستیک نشان داد که درک کنترل رفتار (001/0P<)، قصد رفتار (001/0P<) و هنجار‌های انتزاعی (01/0P<) پیش‌بینی‌کننده‌های معنادار رفتار ماموگرافی می‌باشند. نتیجه‌گیری: با توجه به نتایج به دست آمده از این مطالعه عوامل پیش‌بینی‌کننده انجام ماموگرافی بر اساس تئوری رفتار برنامه‌ریزی شده، هنجارهای انتزاعی، قصد و ادراک کنترل رفتار می‌باشند و هر زمان که زنان ماموگرافی را به عنوان یک هنجار تأیید شده تلقی کنند و قصد انجام آن را داشته و انجام آن را تحت کنترل خود بدانند، ماموگرافی را انجام خواهند داد .

Title: بررسی تاثیر مداخله آموزشی ترکیبی بر پیشگیری از اعتیاد به اینترنت در دانشجویان دختر ساکن خوابگاه‌های علوم پزشکی تهران : کاربرد مدل اعتقاد بهداشتی

http://hsr.mui.ac.ir/index.php/jhsr/article/view/1211

مقدمه: با توجه به شیوع بالای اعتیاد به اینترنت در بین دانشجویان و عوارض بي‌شمار آن و نظر به اهمیت اجرای مداخلات آموزشی مبتنی بر تئوری در پیشگیری و کاهش اختلالات رفتاری از جمله اعتیاد به اینترنت، پژوهش حاضر با هدف تعیین تاثیر مداخله آموزشی ترکیبی بر پیشگیری از اعتیاد به اینترنت در دانشجویان دختر ساکن خوابگاه‌های علوم پزشکی تهران مبتنی بر سازه‌های مدل اعتقاد بهداشتی طراحی و اجرا گردید. روش‌ها: مطالعه حاضر از نوع مطالعات نیمه تجربی قبل و بعد دو گروهی بود که باروش نمونه‌گیری خوشه‌ای چند مرحله‌ای با حجم نمونه80 نفر در سال 1391-1392 انجام شد. ابزار گردآوری اطلاعات شامل پرسشنامه چند‌وجهی اطلاعات دموگرافیک و مرتبط به خصوصیات افراد در مورد اینترنت، ابزار استاندارد اختلال اعتیاد به اینترنت یانگ و پرسشنامه محقق ساخته مبتنی بر سازه‌های مدل اعتقاد بهداشتی بود. روایی و پایایی پرسشنامه ازطریق روایی محتوا و آزمون - آزمون مجدد تعیین شد. مداخله آموزشی ترکیبی در خصوص آشنایی با عوامل و عوارض اعتیاد اینترنتی و برگزاری نمایشگاه پیام‌های بهداشتی با استفاده از پوسترهای طراحی شده توسط محققین انجام شد. داده‌ها با نرم افزار  SPSSنسخه 16تجزیه و تحلیل شد. سطح معنی‌داری در این مطالعه کمتر از 05/0 در نظر گرفته شد. یافته‌ها: میزان پاسخگویی 100٪ بود. میانگین سنی افراد شرکت‌کننده6 /2± 22 سال بود. متغیر وضعیت تاهل با اعتیاد به اینترنت ارتباط معنی‌دار آماری داشت (03/0=P). نتایج حاصل از این پژوهش نشان داد که میانگین امتیاز سازه‌های مدل اعتقاد بهداشتی و آگاهی قبل و چهار ماه بعد ازمداخله آموزشی تفاوت آماری معناداری داشت( 001/0P<) . از طرف دیگر شیوع اعتیاد به اینترنت از2/26 قبل از مداخله آموزشی به 8/13بعد از مداخله آموزشی کاهش یافت( 001/0P<). نتیجه‌گیری: آموزش بر مبنای مدل اعتقاد بهداشتی در ارتقا آگاهی و عملکرد دانشجویان در رابطه با پیشگیری از اعتیاد به اینترنت اثربخش بود. نتایج این مطالعه پیشنهاد می‌کند که مداخلات آموزشی مبتنی بر تئوری‌های آموزش بهداشت و ارتقا سلامت می‌تواند در پیشگیری و کاهش اعتیاد به اینترنت موثر باشد.

Title: به کارگیری مدل تضمین آموزش در بررسي آگاهي و نگرش اعضای هيأت علمي دانشگاه علوم پزشكي اصفهان در مورد طرح درس

http://hsr.mui.ac.ir/index.php/jhsr/article/view/1166

مقدمه: آموزش علوم پزشكي مستلزم وجود برنامه آموزشي كارآمد می‌باشد و اهمیت طرح درس در پیشبرد آموزش انکارناپذیر است. این مطالعه با هدف بکارگیری مدل تضمین آموزش در بررسي آگاهي و نگرش اعضای هيأت علمي دانشگاه علوم پزشكي اصفهان در مورد طرح درس در سال 1392 انجام شد. روش‌ها: تحقيق حاضر از نوع مطالعات توصيفي- تحليلي مي‌باشد که جامعه آماري مطالعه حاضر شامل كليه اعضاي هيأت علمي دانشگاه علوم پزشكي اصفهان نفر بودند که به صورت سرشماري وارد مطالعه شدند ابزار گردآوري اطلاعات شامل پرسشنامه محقق ساخته در سه بخش شامل سؤالات دموگرافیک، سؤالات آگاهی (37 سؤال چهارگزینه‌ای) در قالب اجزا مدل تضمین آموزش و سؤالات نگرشی بود که روایی صوری، محتوی و پایایی آن مورد تایید قرار گرفت. براي تحليل داده‌ها، از آزمون‌های استنباطي نظیر ضريب همبستگي پیرسون و آزمون تحليل واريانس يك عاملي و T-test استفاده شد. داده‌ها با استفاده از نرم‌افزار آماري SPSS نسخه 18 مورد تجزيه تحليل قرار گرفتند. یافته‌ها: میانگین نمره کل اعضای هیأت علمی شرکت‌کننده در مطالعه 14 (از 37) با انحراف معیار 2/8 بود در آیتم‌های اصول برنامه‌ریزی، محتوی و اهداف آموزشی اختلاف معنی‌داری بین اعضای هیأت علمی دانشکده‌ها وجود داشت. بیشترین نمره کل آگاهی به اعضای هیأت علمی با تحصیلات فوق تخصص و کمترین نمره به سطح تحصیلات تخصص اختصاص داشت و اعضای هیأت علمی مرد و زن به ترتیب کمترین و بیشترین نمره کل آگاهی را کسب کردند. در تمام آیتم‌های اجزا مدل تضمین آموزش اختلاف معنی‌داری بین اعضای هیأت علمی بر حسب سابقه کارگاه وجود داشت. میانگین نمره کل نگرشی اعضای هیأت علمی شرکت‌کننده در مطالعه 6/44 با انحراف معیار 4/3 بود. نتیجه گیری: اساتید دانشگاه از نظر آگاهی در وضعیت مناسبی قرار نداشتند اما نگرش نسبتا خوبی به طرح درس داشتند لزوم برنامه‌ریزی‌های دقیق و مداوم برای بهبود این مسأله ضروری است.

Title: بررسی ایزوترم و سینتیک جذب سرب و کادمیوم از شيرابه زباله توسط جاذبهای طبیعی

http://hsr.mui.ac.ir/index.php/jhsr/article/view/1147

مقدمه: حضور فلزات سنگين در شرابه به دليل توانايي تجمع پذيري زيستي سلامت موجودات زنده به خصوص انسان را با خطرات بسياري مواجه مي نمايد. هدف از اين تحقيق بررسي امکان جذب دو فلز سرب و کادميوم از شیرابه به وسيله زئوليت طبيعي کلينوپتيلوليت و امکان افزايش توانايي جذب اين زئوليت توسط اصلاح سطح ان به کمک سورفكتانت HDTMA-Br مي باشد. روشها:در اين مطالعه از زئوليت طبيعي کلينوپتيلوليت به دوصورت اصلاح نشده (NMZ) و اصلاح شده (SMZ) توسط سورفکتانت هگزادسيل تري متيل آمونيوم-برومايد (HDTMA-Br) جهت جذب فلزات سرب و کادميوم استفاده شد. جهت بررسي توان جذب اين دوفلز، متغييرهاي چون نوع جاذب، غلظت جاذب و زمان تماس مورد بررسي قرار گرفتند. نمونه زئوليت استفاده شده توسط آزمايشات X-ray Florescence، طيف X-ray diffraction وBET جهت شناسايي ترکيب مورد آناليز قرار گرفت. يافته ها: اصلاح زئوليتها باعث افزايش راندمان جذب به ترتيب از2/4 و 3/5 درصد به 64/71 و 2/75 درصد گردید. با افزايش غلظت سورفکتانت از 2 به 20 میلی مول بر لیتر راندمان جذب سرب و کادميوم به ترتيب 71 و 74 درصد افزايش يافت. همچنين مشاهده شد راندمان جذب با افزايش زمان تماس و افزايش نسبت جاذب به محلول، رابطه مستقيم دارد. نتيجه گيري: اصلاح زئوليتها باعث افزايش چشمگير جذب فلزات سنگين از ترکيب شيرابه گردید. سرب و کادمیوم به صورت تک لایه ای بر روی زئولیتهای اصلاح شده با سورفکتانت جذب شده و مقایسه مدل های ایزوترم نشان می دهد که ظرفیت زئولیتهای اصلاح شده در جذب سرب بیشتر از کادمیوم بوده ولی کادمیوم با انرژی بیشتری جذب می شود.

Title: بررسی غلظت برخی از فلزات سنگین درآب و رسوب رودخانه تمبی مسجد سلیمان قبل و بعد از ورود فاضلاب به آن

http://hsr.mui.ac.ir/index.php/jhsr/article/view/1139

مقدمه :این مطالعه به منظور بررسی غلظت فلزات سنگین مس، روی، سرب، کروم، کادمیوم، نیکل و کبالت در آب و رسوب رودخانه تمبی واقع در استان خوزستان، شهر مسجد سلیمان قبل و بعد از ریزش فاضلاب (منظور فاضلاب تصفیه نشده شهر، پساب کشتارگاه سنتی ، شیرابه حاصل از زباله‌های تجمع یافته در حاشیه رودخانه و روان آب‌های سطح شهر می‌باشد) به آن انجام شد. روش‌ها: این تحقیق یک مطالعه تجربی- توصیفی می‌باشد که در آن نمونه‌های آب و رسوب به مدت یک سال از دو نقطه بالادست و پایین دست محل ورود فاضلاب  به رودخانه تمبی برداشته شدند .نمونه‌ها در آزمایشگاه، خشک، تغلیظ و هضم گردیدند و غلظت فلزات سنگین در آنها به وسیله دستگاه جذب اتمی شعله‌ای اندازه‌گیری شد.  یافته‌ها: نتایج نشان دادند که میانگین غلظت فلزات مورد نظر در آب و رسوب پایین دست نسبت به بالا دست در فصول مختلف بیشتر است. غلظت‌های به دست آمده برای مس و روی در محدوده استاندارد آب آشامیدنی، میانگین غلظت منگنز، سرب و روی در محدوده استاندارد آب برای کشاورزی و میانگین غلظت کادمیوم، کرم، سرب و نیکل از استاندارد آب‌های سطحی بیشتر می‌باشد. بیشترین میانگین غلظت مربوط به سرب درآب پایین دست برابرppm 09/0  95/1و منگنز در رسوب پایین دست برابر  ppm5/2 820 و کمترین میانگین غلظت مربوط به کادمیوم در آب و رسوب بالادست به ترتیب برابر ppm (001/0  07/ 0و 5/0 10) می‌باشد. میانگین غلظت فلزات سنگین در رسوب پایین دست نسبت به رسوب بالادست افزایش یافته است. نتیجه‌گیری: با توجه به نتایج حاصل، استفاده ازآب رودخانه تمبی برای اهداف تفریحی، شستشو، ماهیگیری و برای سلامت انسان و محیط زیست مناسب نمی‌باشد و لازم است که نسبت به کنترل فاضلاب و تصفیه آن قبل از ورود به این رودخانه اقدامات جدی صورت گیرد و کیفیت آب و رسوبات این رودخانه متناسب با اهداف بهره‌برداری مدیریت شود.

Title: بررسی عوامل زمینه‌ساز عفونت در کانولاهای وریدی محیطی از دیدگاه پرستاران در بخش‌های داخلی‌جراحی بیمارستان‌های منتخب دانشگاه علوم پزشکی اصفهان

http://hsr.mui.ac.ir/index.php/jhsr/article/view/1131

مقدمه :به منظور رسانيدن آب و مايعات و تأمين الکترولیت‌ها و انجام اقدامات طبي در بيماران بستري، داشتن يک راه وريدي ضروري است که يکي از روش‌هاي شايع تهاجمي نصب کاتتر داخل وريدي مي‌باشد. اين روش عليرغم ارزش‌درماني يکي از عوامل ايجاد‌کننده عفونت‌هاي بيمارستاني است. هدف از این مطالعه تعیین عوامل زمینه‌ساز ‌عفونت در کانولاهای‌ وریدی محیطی از دیدگاه پرستاران در بخش‌های‌ داخلی و جراحی بیمارستان‌های منتخب دانشگاه‌ علوم‌ پزشکی‌اصفهان می‌باشد. روش‌ها :این پژوهش یک مطالعه توصیفی بود که به صورت مقطعی انجام شد. تعداد 379 پرستار از سه مركز آموزشي‌درماني (الزهرا، نور، عیسی‌بن‌مریم) به صورت تصادفی ساده انتخاب شدند. جهت گرد‌آوری داده‌ها از پرسشنامه خودساخته دو ‌قسمتی شامل مشخصات فردی و عوامل زمینه‌ساز عفونت، استفاده گردید. جهت تجزيه ‌‌و تحليل اطلاعات از روش‌هاي ‌آماري توصيفي ‌و تحلیلی با استفاده از نرم‌افزار آماری  SPSSنسخه 18 استفاده شد. یافته‌ها :اکثریت نمونه‌های مورد پژوهش مونث (6/80%) و محدوده سنی آن‌ها 22 تا 52 سال بود. بیشترین درصد فراوانی عوامل زمینه‌ساز عفونت در کانولاهای وریدی محیطی از دیدگاه پرسنل پرستاری در هر سه بیمارستان به ترتیب، مربوط به شستن دست‌ها قبل از انجام پروسیجر (100%)، استفاده از دستکش (2/99%)، ضدعفونی صحیح محل ورود آنژیوکت (97%)، واردکردن آنژیوکت در محل سالم پوست (97%)، شیو موی ناحیه ورود آنژیوکت (7/95%)، تعویض به موقع آنژیوکت (5/94%)، رعایت اصول استرلیتی در زمان وارد و خارج کردن آنژیوکت (94%) می‌باشد. بر اساس نتایج آزمون اسپیرمن، ارتباط آماری معنی‌داری بین نگرش نسبت به کنترل عفونت با سن (001/0P=، 48/0- r=) و سابقه کار (02/0 P=، 55/0r=) وجود داشت. همچنین آزمون من‌ویتنی نشان داد بین جنسیت با نگرش به کنترل عفونت ارتباط آماری معنی‌دار وجود دارد (04/0P=). نتيجه‌گيري: یافته‌های این مطالعه بر اهمیت و الزام توجه دقیق‌‌تر و عمیق‌تری نسبت به عوامل زمینه‌ساز عفونت در راستای کاهش و کنترل عفونت‌های ناشی از کاتترهای وریدی محیطی صحه می‌گذارد.

Title: بررسی موانع درک شده انجام فعالیت جسمانی در دانش‌آموزان دختر و پسر دبیرستانی شهرستان ایذه در سال 1391- 1390

http://hsr.mui.ac.ir/index.php/jhsr/article/view/1089

مقدمه: مطالعات زیادی در کشورهای مختلف دنیا به منظور شناسایی موانع انجام فعالیت جسمانی در نوجوانان و جوانان انجام شده است. اما در ارتباط با این موضوع در کشور ایران اطلاعات  کافی در دسترس نیست. لذا این مطالعه با هدف جبران این نقیصه، در دانش‌آموزان دبیرستانی شهرستان ایذه انجام شد.                                                                                                          روش‌ها: در این مطالعه توصیفی تحلیلی تعداد 500 دانش‌آموز دبیرستانی (شامل 300 دختر و 200 پسر) به روش نمونه‌گیری تصادفی چند مرحله‌ای انتخاب شدند. سپس با استفاده از یک پرسشنامه، موانع انجام فعالیت فیزیکی شرکت‌کنندگان مورد ارزیابی قرار گرفت. پس از تکمیل پرسشنامه‌ها، داده‌ها جمع‌آوری و با استفاده از برنامه SPSSنسخه 18 تجزيه و تحليل شد. ملاک معناداری آماري 05/0 P<بود.                                                               یافته‌ها : 8/69 % کل دانش‌آموزان غیرفعال بودند. دانش‌آموزان پسر نسبت به دختران فعال‌تر بودند. موانع خارجی انجام فعالیت جسمانی نسبت به موانع داخلی از اهمیت بیشتری برای نوجوانان برخوردار بودند. 3 سؤال «مکان‌های ورزشی کمی در اختیار من است »، «برنامه زمان‌بندی اماکن ورزشی موجود، با شرایط من هماهنگ نیست» و «هزینه‌های ورزش خیلی زیادند» به عنوان مهمترین موانع انجام فعالیت فیزیکی توسط دانش‌آموزان ذکر شدند.                                                                                                                                                                                 نتیجه گیری: نتايج اين مطالعه نشان داد موانع خارجی نسبت به موانع داخلی انجام فعاليت جسماني در دانش‌آموزان نقش موثرتری دارد. همچنین دانش‌آموزان دختر بیشتر از پسران موانع را عامل مهم جلوگیری از انجام فعالیت جسمانی ذکر کردند. لذا درك اين عوامل به آموزش‌دهندگان و متخصصين سلامت و ساير مسؤولین در طراحي برنامه‌هاي مداخلاتي جهت ارتقای فعالیت جسمانی نوجوانان كمك می‌کند.

Title: بررسی تاثیر ریسک فاکتورهای زیستی و روانی مادران بر کم وزنی نوزادان در استان اصفهان

http://hsr.mui.ac.ir/index.php/jhsr/article/view/965

مقدمه: یکی از شاخص‌های بهداشتی مهم در بهداشت عمومی، ویژگی‌های اولیه نوزاد مخصوصا وزن هنگام تولد است. هدف این مطالعه بررسی تاثیر ویژگی‌های زیستی-روانی مادران بر کم وزنی هنگام تولد در استان اصفهان بود. روش‌ها: این پژوهش در سال 1388 بر روی 910 نوزاد تازه متولد شده و مادران آنها از استان اصفهان انجام شد. برای انتخاب نمونه‌ها از روش نمونه‌گیری طبقه‌ای و خوشه‌ای با توجه به نسبت جمعیت در هر یک از شهرستان‌های استان اصفهان استفاده شد. اطلاعات با پرسش‌نامه ویژگی‌های زیستی، روانی و اجتماعی والدین کودکان استثنایی و آزمون رضایت‌مندی زناشویی اینریچ جمع‌آوری شد. داده‌ها با آماره‌های توصیفی و تحلیل رگرسیون لجستیک نرم افزار SPSS نسخه 16 تحلیل شدند. یافته‌ها: یافته‌ها نشان داد که فراوانی نوزادان کم وزن ( وزن کمتر از 2500 گرم) در استان اصفهان 5/9٪ بود. فراوانی بارداری‌های ناخواسته 7/38٪ ، 6/15٪ بارداری‌ها زیر 20 سال و 22٪ بالای 35 سال بود. 9/38٪ از مادران در دوران بارداری در معرض دود سیگار بودند. میانگین افزایش وزن دوران بارداری 9کیلوگرم بود.33٪ مادران در دوران بارداری فشار خون بالا، 7/26٪ سابقه‌ی سقط جنین و 9/31٪ سابقه خون‌ریزی داشتند 1/21٪ والدین نسبت خویشاوندی نزدیک داشتند و 7/29٪ زایمان‌ها به صورت سزارین بود. تحلیل رگرسیون لجستیک نشان داد که نارضایتی زناشویی، فشار خون غیرطبیعی، اشتغال در دوران بارداری، افزایش وزن کمتر از 5 کیلوگرم، قد مادر، حاملگی زیر 20سالگی مسمومیت وخون‌ریزی دوران بارداری در دوران بارداری و با کم وزنی هنگام تولد ارتباط معناداری وجود دارد. نتیجه‌گیری: نتایج این مطالعه نشان داد که برخی از ویژگی‌های زیستی و روانی مادران بر کم وزنی هنگام تولد تاثیر قابل توجهی دارند و می‌تواند در برنامه‌های پیشگیری از تولد نوزادان کم وزن در استان اصفهان مورد توجه بیشتری قرار گیرد.
